# 702. Efficacy of Fecal Microbiota, Live-jslm After Receipt of Non–Clostridioides difficile Infection Antibiotics: Post Hoc Subgroup Analysis of a Phase 2 Open-Label Trial

**DOI:** 10.1093/ofid/ofad500.764

**Published:** 2023-11-27

**Authors:** Kelly R Reveles, Anne J Gonzales-Luna, Yoav Golan, Carolyn D Alonso, Beth Guthmueller, Xing Tan, Monique Bidell, Victoria Pokhilko, Carl Crawford, Andrew M Skinner

**Affiliations:** The University of Texas at Austin, San Antonio, Texas; Department of Pharmacy Practice and Translational Research, University of Houston College of Pharmacy, Houston, Texas, USA, Houston, TX; Tufts Medical Center, Boston, Massachusetts; Beth Israel Deaconess Medical Center, Boston, Massachusetts; Rebiotix Inc., a Ferring Company, Roseville, Minnesota; Ferring Pharmaceuticals, Parsippany, New Jersey; Ferring Pharmaceuticals, Parsippany, New Jersey; Ferring Pharmaceuticals, Parsippany, New Jersey; Weill Cornell Medicine, New York, New York; University of Utah, Holladay, Utah

## Abstract

**Background:**

Live biotherapeutic products (LBPs) are adjunctive therapies to prevent recurrence of *Clostridioides difficile* infection (CDI). In this post hoc subgroup analysis, the durability of fecal microbiota, live-jslm (REBYOTA™; abbreviated here as RBL, previously known as RBX2660), the first microbiota-based LBP approved by the US Food and Drug Administration for the prevention of recurrent CDI (rCDI) in adults following antibiotic treatment for rCDI, was investigated in a subset of participants who received systemic antibiotics for indications other than CDI following RBL treatment in a phase 2 open-label trial (NCT02589847).

**Methods:**

PUNCH Open Label participants were ≥ 18 years old with either ≥ 2 rCDI episodes treated with standard-of-care antibiotic therapy after a primary CDI episode, or ≥ 2 severe CDI episodes requiring hospitalization. Participants in this analysis all received 2 doses of RBL rectally administered approximately 7 days apart. We report outcomes at various timepoints for participants who were successfully treated with RBL and subsequently received non-CDI systemic antibiotics.

**Results:**

A total of 43 participants were included in the analysis; the mean age was 65.9 years, most were male (67.4%), and most (97.7%) had ≥ 3 rCDI episodes before RBL. Most participants were treated with antibiotic monotherapy (n=34/43, 79.1%), received 1 course of treatment (n=28/43, 65.1%), and did not receive CDI prophylaxis (n=38/43, 88.4%). The median time to antibiotic exposure after the second dose of RBL was 155 days (interquartile range [IQR], 55-349), and the median duration of treatment per antibiotic course was 8 days (IQR, 4.5-12.5). Urinary system infections were the most common indication for antibiotic treatment (n=17/43, 40%). Among evaluable participants who received systemic antibiotics within 8 weeks, 6 months, 12 months, and 24 months of RBL administration, 91.6% (11/12), 95.7% (22/23), 90.6% (29/32), and 83.3% (30/36) remained CDI recurrence-free, respectively. A total of 86% (37/43) of participants were recurrence-free at their last evaluable timepoint.

**Conclusion:**

In this post hoc analysis, RBL remained effective in preventing CDI recurrence in patients with multiple episodes of rCDI after subsequent systemic antibiotic exposure.

**Disclosures:**

**Kelly R. Reveles, PharmD, PhD**, Ferring Pharmaceuticals: Advisor/Consultant|Ferring Pharmaceuticals: Honoraria **Anne J. Gonzales-Luna, PharmD, BCIDP**, Cidara Therapeutics: Grant/Research Support|Ferring Pharmaceuticals: Personal Fees|Paratek Pharmaceuticals: Grant/Research Support|Seres Therapeutics: Grant/Research Support **Yoav Golan, MD**, Ferring Pharmaceuticals: Advisor/Consultant|Pfizer: Honoraria|Seres Therapeutics: Advisor/Consultant|Vedanta Bioscience: Advisor/Consultant **Carolyn D. Alonso, MD**, Academy for Continued Healthcare Learning: Honoraria|AiCuris: Advisor/Consultant|American Society of Healthcare Pharmacists: Honoraria|Cidara Therapeutics: Advisor/Consultant|Clinical Care Options: Honoraria|Merck: Advisor/Consultant|Merck: Grant/Research Support **Beth Guthmueller, AS**, Rebiotix Inc., a Ferring Company: Employee **Xing Tan, PharmD**, Ferring Pharmaceuticals: Employee **Monique Bidell, PharmD**, Ferring Pharmaceuticals: Employee **Victoria Pokhilko, PhD**, Ferring Pharmaceuticals: Employee **Carl Crawford, MD**, Ferring Pharmaceuticals: Advisor/Consultant **Andrew M. Skinner, MD**, Academy for Continued Healthcare Learning: Honoraria|American Society of Healthcare Pharmacists: Honoraria|Ferring Pharmaceuticals: Honoraria|MJH Life Sciences: Honoraria

